# Supplementary material for: Win Statistics in Observational Cancer Research: Integrating Clinical and Quality-of-Life Outcomes
Source: J Clin Med. 2024 May 31;13(11):3272. doi: 10.3390/jcm13113272 (PMC11173121; doi:10.3390/jcm13113272)
Supplement: Supplementary file 1 [file jcm-13-03272-s001.zip › jcm-3015037-supplementary.pdf]

Supplementary Material

Table S1. Baseline characteristics among Treated (T) and Control (C) groups, in observational setting at difference imbalance degrees (simulation study)

**Characteristic**	**C, N = 100[1]**	**T, N =100[1]**	**p-value[2]**
**Imbalance: high**			
**X1,** dichotomous	44%	61%	0.016
**X2,** continuous	54.6 (50.9-58.5)	48.1 (44.9-51.2)	<0.001
**X3,** dichotomous	46%	61%	0.033
**Imbalance: medium**			
**X1,** dichotomous	44%	57%	0.07
**X2,** continuous	54.6 (50.9-58.5)	50.1 (46.9-53.2)	<0.001
**X3,** dichotomous	46%	60%	0.047
**Imbalance: low**			
**X1,** dichotomous	44%	54%	0.2
**X2,** continuous	54.6 (50.9-58.5)	52.1 (48.9-55.2)	0.002
**X3,** dichotomous	46%	50%	0.09

[1] n (%); Median (IQR); [2] Pearson's Chi-squared test, Wilcoxon rank sum test, Fisher's exact test.

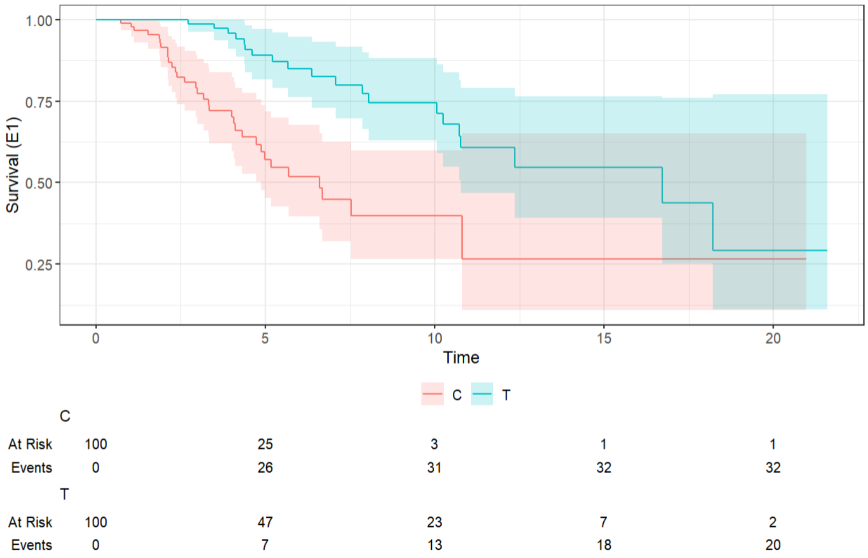

Figure S1. Kaplan-Meier curves for E1, RCT setting.

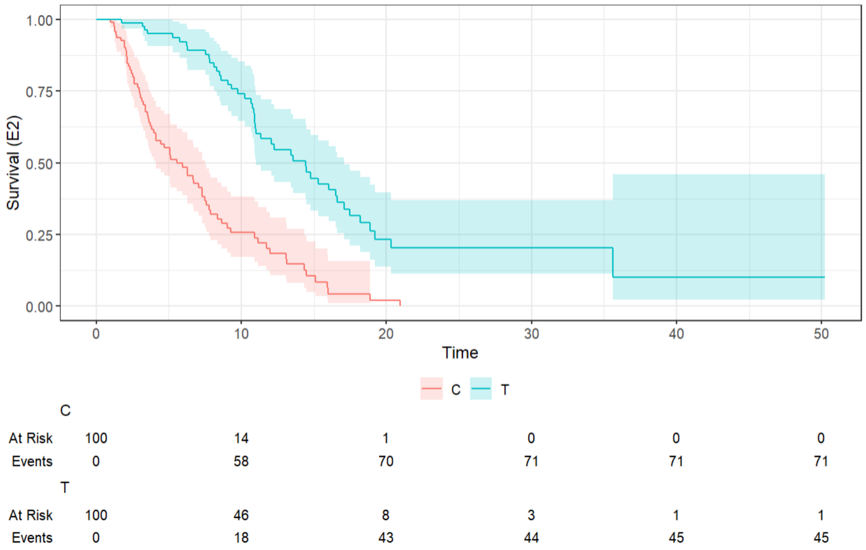

Figure S2. Kaplan-Meier curves for E2, RCT setting.

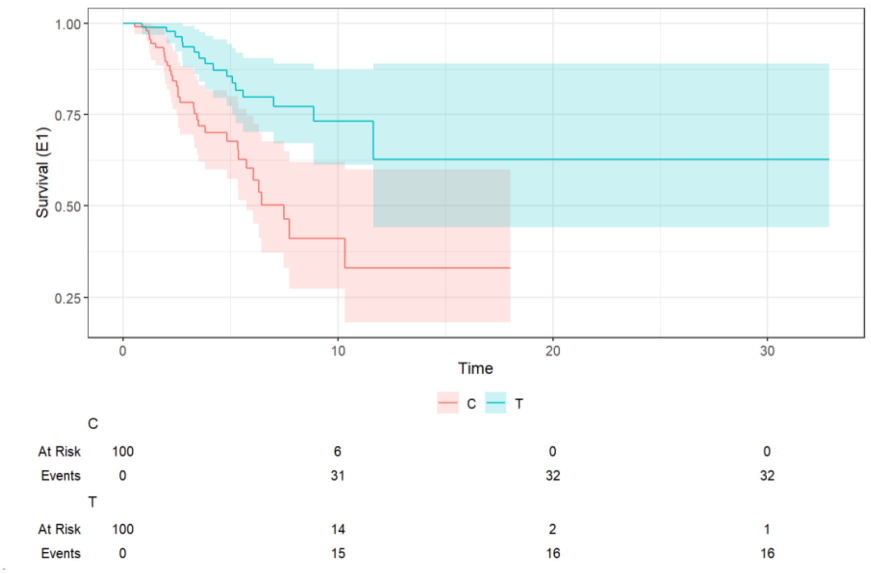

Figure S3. Kaplan-Meier curves for E1, observational, low imbalance.

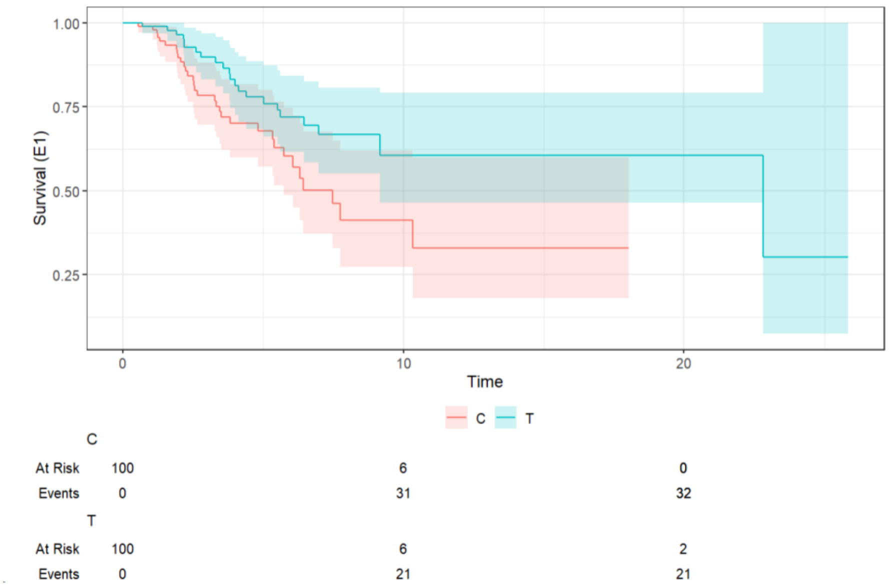

Figure S4. Kaplan-Meier curves for E1, observational, medium imbalance.

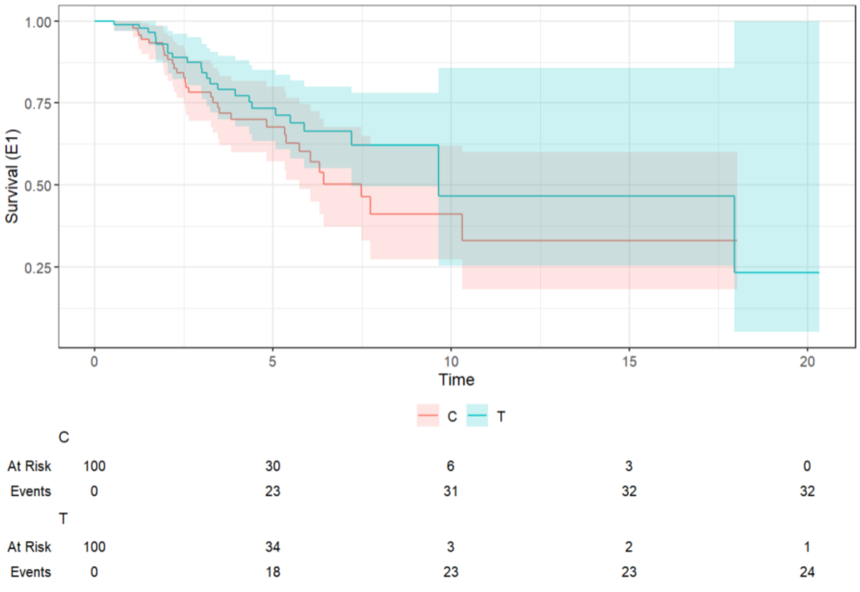

Figure S5. Kaplan-Meier curves for E1, observational, high imbalance.

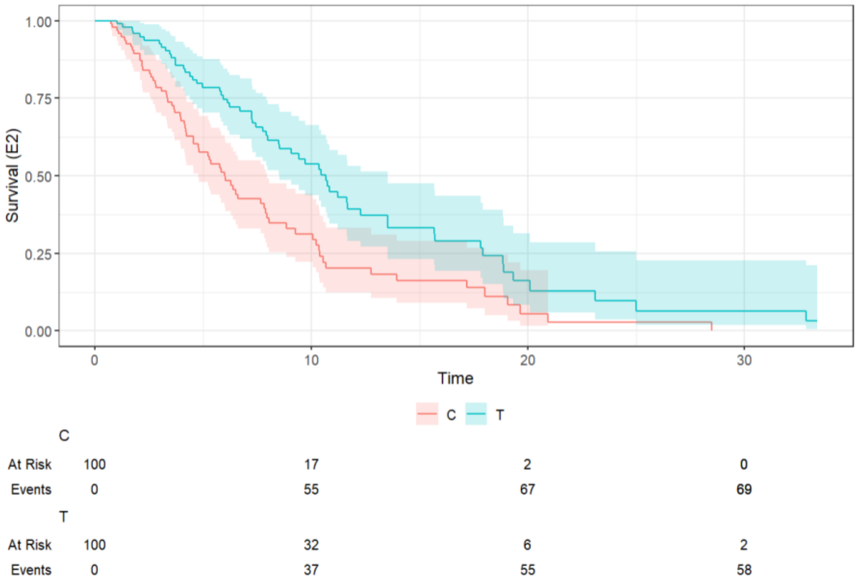

Figure S6. Kaplan-Meier curves for E2, observational, low imbalance.

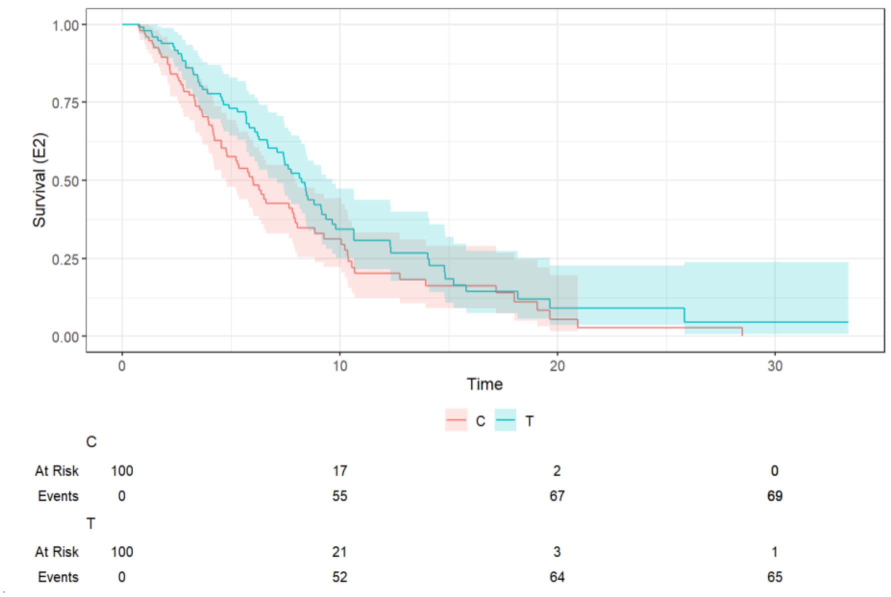

Figure S7. Kaplan-Meier curves for E2, observational, medium imbalance.

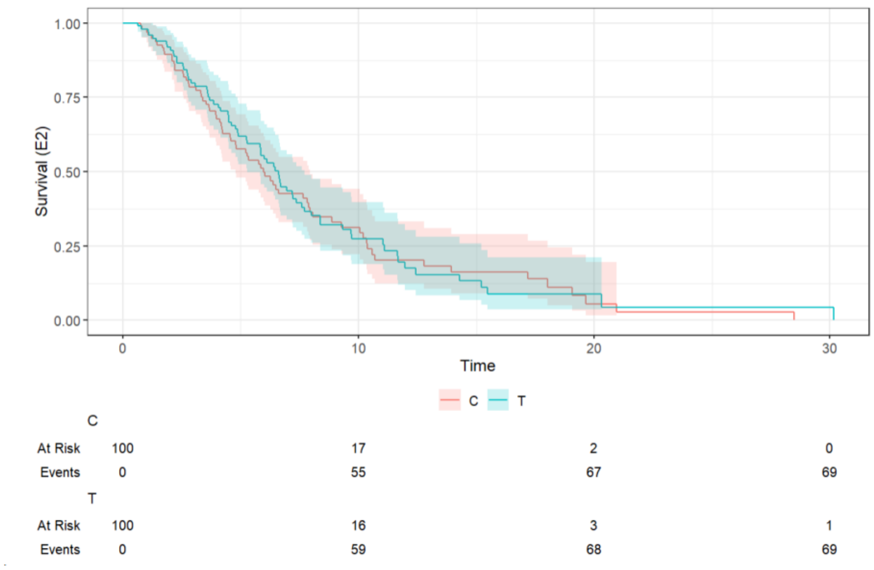

Figure S8. Kaplan-Meier curves for E2, observational, high imbalance.
